# Supplementary figures and images for: miQC: An adaptive probabilistic framework for quality control of single-cell RNA-sequencing data
Source: PLoS Comput Biol. 2021 Aug 24;17(8):e1009290. doi: 10.1371/journal.pcbi.1009290 (PMC8415599; doi:10.1371/journal.pcbi.1009290)

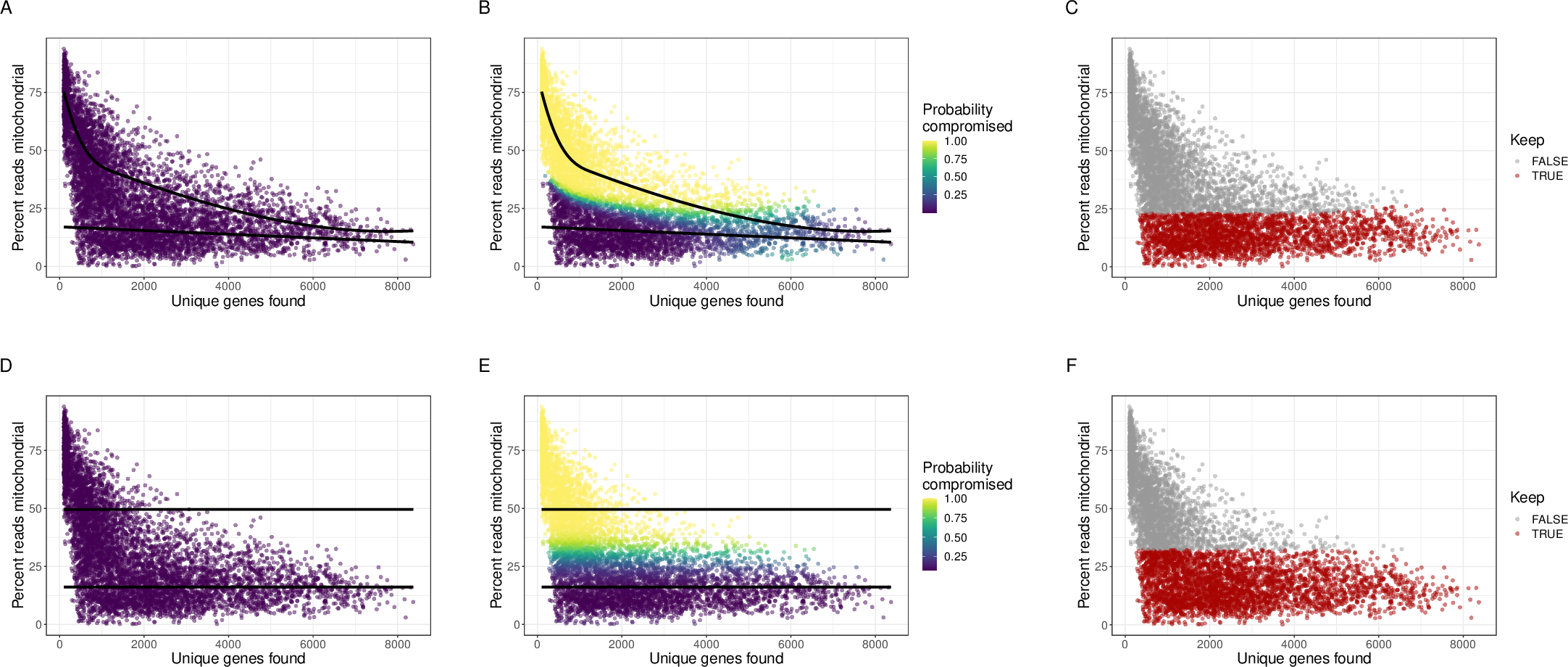

Supplement: S1 Fig — (A) A mixture model on high-grade serous ovarian tumor data, where the intact cell distribution is modeled linearly and the compromised cell distribution is modeled using a b-spline.(B) Posterior probability of tumor cells belonging to compromised distribution as fitted with a spline model. (C) Cells with greater than 75% posterior probability of being compromised are marked for removal, after the two default corrections (keep_all_below_boundary = TRUE and enforce_left_cutoff = TRUE). (D-F) The same tumor, but with a one-dimensional Gaussian mixture model on percent mitochondrial reads. (TIF) [file pcbi.1009290.s001.tif]

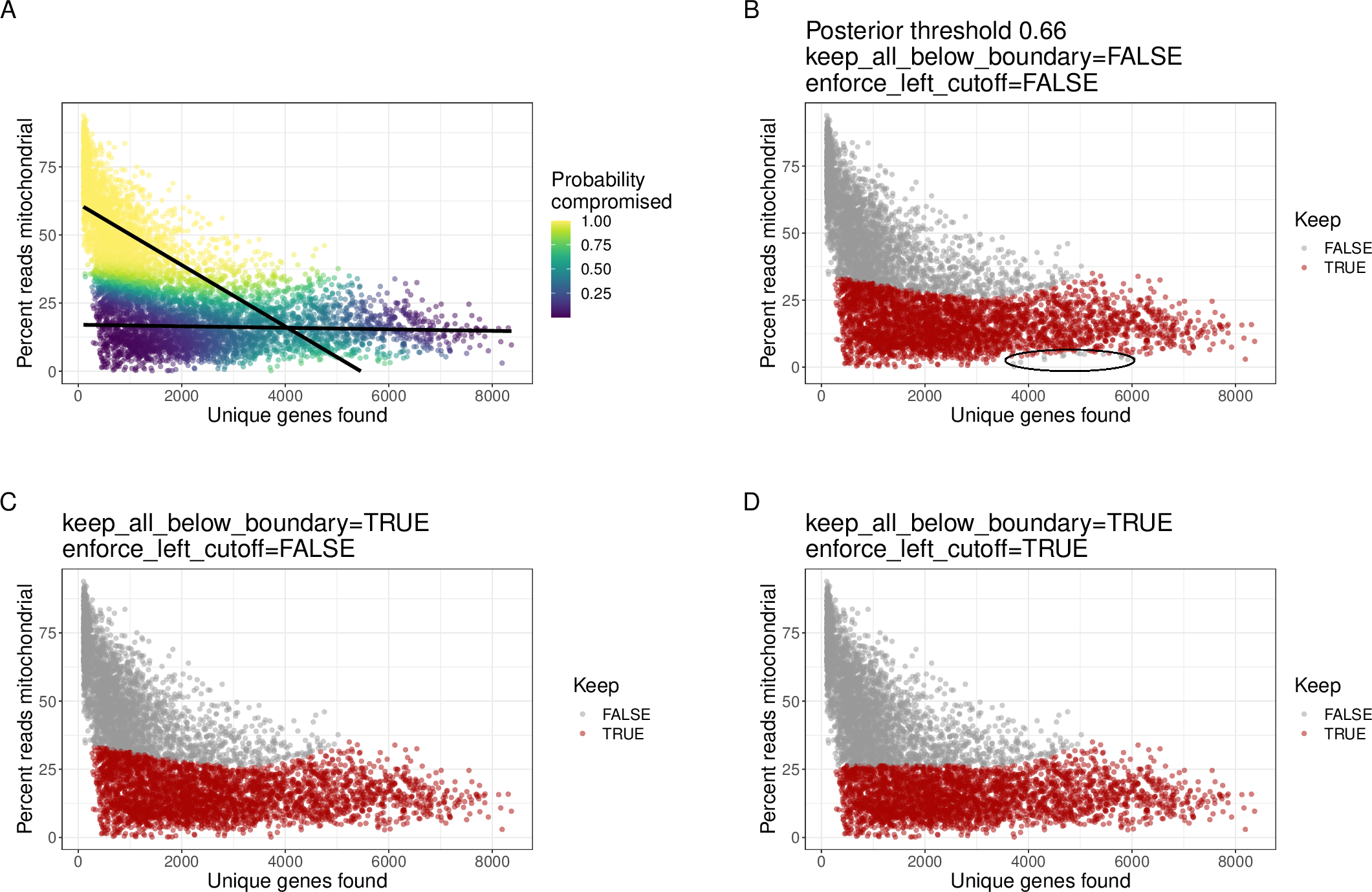

Supplement: S2 Fig — (A) The posterior distribution of linear mixture models from Fig 1, shown again for reference. (B) Filtering based only using a 66% posterior threshold with no default corrections (keep_all_below_boundary = FALSE and enforce_left_cutoff = FALSE) Low-mitochondrial cells slated for removal are circled in black. (C) Same as (B), but including the keep_all_below_boundary = TRUE parameter, where all cells with lower mitochondrial fraction than the predicted intact distribution are kept. This prevents the group of cells in the bottom center from being excluded. (D) Same as (B), but including both default corrections (keep_all_below_boundary = TRUE and enforce_left_cutoff = TRUE). This corrects the U-shape boundary. The excluded cell with the lowest mitochondrial percentage is identified, and any cell with both greater mitochondrial percentage and lower library complexity is excluded. (TIF) [file pcbi.1009290.s002.tif]

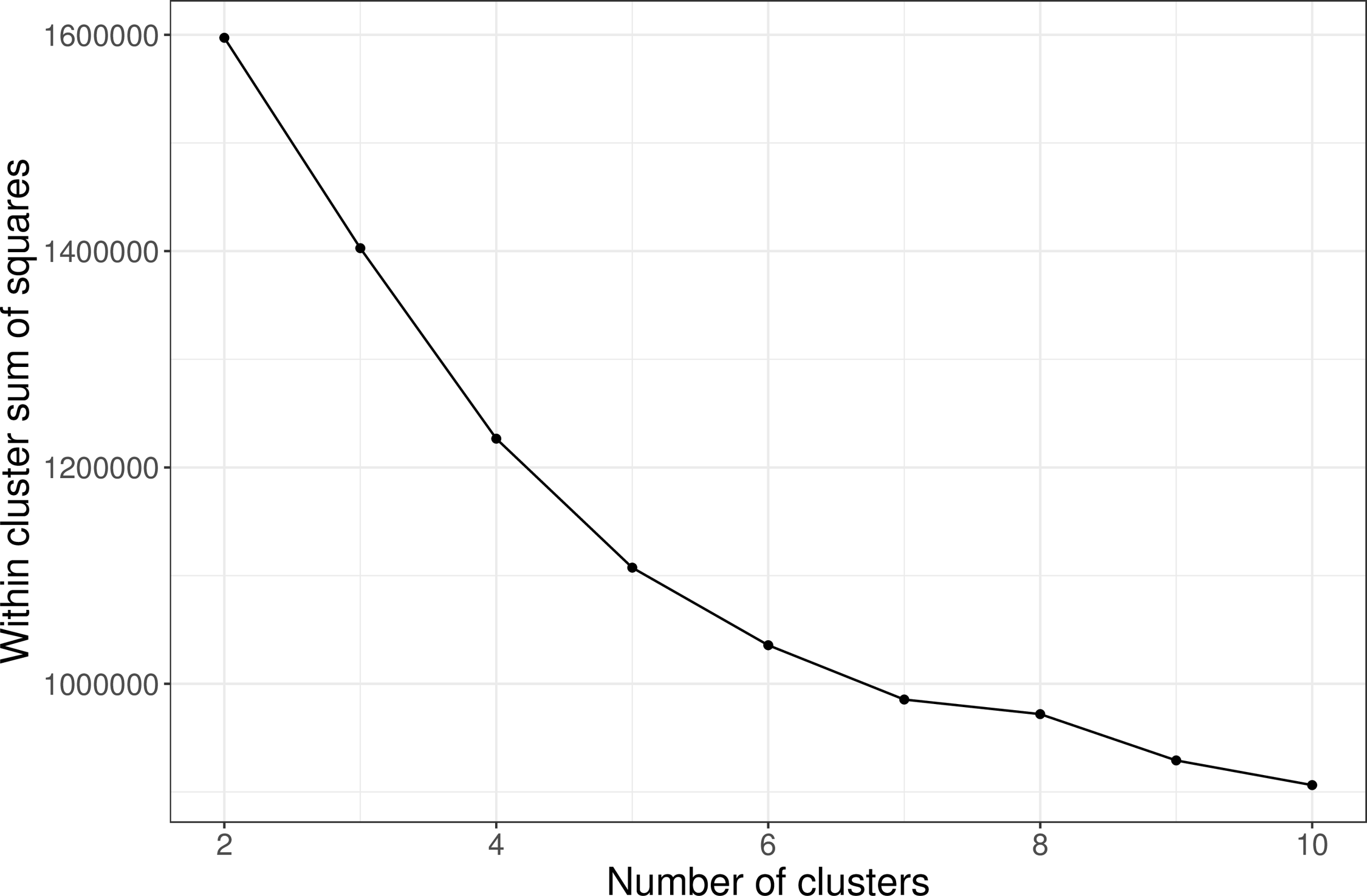

Supplement: S3 Fig — We ran mbkmeans on our tumor data for a range of k from 2 to 10. Based on the within cluster sum of squares (WCSS), we proceeded with 6 clusters. (TIF) [file pcbi.1009290.s003.tif]
