# Supplementary material for: miQC: An adaptive probabilistic framework for quality control of single-cell RNA-sequencing data
Source: PLoS Comput Biol. 2021 Aug 24;17(8):e1009290. doi: 10.1371/journal.pcbi.1009290 (PMC8415599; doi:10.1371/journal.pcbi.1009290)
Supplement: Attachment — Submitted filename: miQC-review.pdf [file pcbi.1009290.s011.pdf]

Summary

In this manuscript, the authors present an adaptive data-driven quality filtering threshold for scRNAseq data, based on two quality measures: the number of unique genes per cell, and the proportion of counts mapping to mitochondrial genes. The aim of this method is to identify "low-quality" cells that may bias the results of downstream analyses. This is a key component of every single-cell analysis, and is perhaps an under-addressed component of the field of single cell computational biology.

To this end, they use a finite mixture of regression models, where one component of the mixture represents "compromised" or low-quality cells, and the other component represents high-quality cells. The posterior probabilities from this mixture model are then used to classify cells as high-quality or low-quality. Using the dependence between the two quality measures, the authors aim to filter cells in a dataset-dependent way that can adapt easily to tissues containing varying numbers of high- and low-quality cells.

The authors make a commendable effort to devise an adaptive, data-driven quality measure for scRNAseq. Furthermore, I commend their presentation of their method as a reviewed Bioconductor software package, as well including the datasets and code used for analysis as a reproducible repository. Their results are easily reproducible and their presentation of these materials is, in my view, exemplary.

The authors place their method well in the context of the wider literature on the topic. Furthermore, their manuscript is well-organised and in general well-written and easily understood. However, at times there appear to exist discrepancies between the motivations they describe for a data-driven quality control measure and the behaviour of their approach on the data they present. This could be clarified by explaining the motivations behind their choice of model, by clarifying the reasoning why cells should be considered high- or low-quality, and/or by adapting their methodology to make it more consistent with their stated aims.

Major comments

While adaptive and data-driven approaches to quality control are both novel and desirable, it is not clear to me the motivation behind this specific approach. I am uncertain why one expects a different relationship between mitochondrial proportion and number of unique genes for "high-quality" and "low-quality" cells, as is implied by a mixture of linear models. It seems to me that the approach was chosen for simplicity based on the desire to identify cells in the top-left of the bivariate plots as low-quality and those in the remainder of the plot as high-quality. I think that this is fine conditional on the model producing results that are consistent with the view of these two measures as markers of cell quality.

However, I think there are some inconsistencies between the stated motivation for an adaptive, data-driven method and how this method functions when applied. I think that these inconsistencies should be addressed before publication, and I hope they may lead the authors to consider adapting their method to address them. To illustrate some of these issues, I have included some PDF documents I generated using the authors' code and datasets. Each of these is was created by running an RMarkdown script using a selection of the datasets in the `mito-filtering` repository linked in the manuscript; primarily those examples used in the manuscript.

In these files, I have applied miQC, and a 2-component Gaussian mixture using only the proportion of mitochondrial counts. By comparing these approaches, I hope to illustrate some of the points I raise here. Figure numbering, captioning, etc is consistent throughout.

In the mixture model presented, the model component with the larger intercept is considered the component representing "compromised" cells, where the authors use the definition:

> "a low-quality cell that is expected to have few unique genes represented and a high mitochondrial fraction".

This definition is consistent with the idea that the authors wish to select cells in the top-left region as "low-quality" and those outside as "high-quality". I will refer here for brevity to the "lower component" as the one representing high-quality cells and to the "upper component" as the one representing compromised cells.

Given the very small slope for the lower component in most examples presented, this lower mixture component is essentially a Gaussian distribution over values of mitochondrial proportion, with a fixed mean for all values of the proportion of genes detected. This seems sensible based on the marginal distribution of the data (Figure 1 in attached PDFs). The upper component, meanwhile, has a non-zero slope, representing a Gaussian with a mean that varies based on the number of unique genes detected. Therefore a natural question is: how does miQC compare to a 2-component Gaussian mixture on the proportion of mitochondrial reads, ignoring cellular complexity?

As seen in Figures 6 and 7 of the attached PDFs, these approaches are broadly similar, due in part to the similarity of the lower (non-compromised) mixture components of each model. Cells in the top-left region have almost identical posterior probability values and there are, in the main, few differences in the cells rejected and accepted by each approach. When differences arise, they do not appear favourable to miQC. miQC in some cases retains more cells with very low unique genes and relatively high MT proportion, while removing more cells with higher unique genes and lower MT proportion (see Figure 7 in `16030X4.pdf`). Often, the decision boundary is slightly U-shaped (eg, Figure 4 in files `16030X2.pdf`, `16030X4.pdf`). This results in miQC giving a lower probability of being compromised to cells with "worse" scores on both of the quality measures being used.

This seems to me to be contrary to the stated motivations for this quality measure. Specifically, on line 84 the authors state:

> "mitochondrial transcript content is expected to be relatively high also for intact ovarian cancer cells."

Given that miQC removes cells with high MT proportion and high complexity, it appears that this would favour the removal of intact ovarian cancer cells with high mitochondrial proportion and high cellular complexity. The behaviour of miQC in these data therefore seems to conflict with the stated goal of retaining cells with higher MT proportion for cancer datasets.

The differences between the approaches are quite apparent when comparing the posterior probabilities between the two approaches (Figure 6 in attached PDFs). The right tail of the upper mixture component produces counterintuitive results. For example, in Figure 2 of `16030X2.pdf` and `16030X4.pdf`, miQC gives a higher probability of being compromised for with $< 10\%$ MT counts and c 4000 unique genes than cells with c. 25% MT counts and < 500 unique genes. Again, it seems counterintuitive that cells with excellent values for both quality measures should be considered probable low-quality cells, while cells with very poor values for both measures are not.

Outliers with very low proportion of mitochondrial reads would likely be considered outliers using this approach - this is visible in Figure 4 of `Messmer.pdf`. Furthermore, I would expect this inconsistency to be magnified if users supply more stringent posterior probability thresholds. Under this condition it could easily be the case that cells rejected by miQC would be located in all regions of the bivariate distribution of these two measures, rather than concentrated solely in the upper left region. For example, in Figure 9 in `16030X2.pdf` and `16030X4.pdf`, it's clear that a more stringent probability threshold of $\frac{2}{3}$ causes cells with very low mitochondrial proportion and relatively high complexity to be rejected (c. 0% and c. 5000, respectively). The decision rule could be adapted for cases such as this by choosing to never reject cells with a proportion of mitochondrial reads that is lower than the intercept of the lower mixture component. However, it seems as though needing to adapt the decision rule after fitting the model points to some deficiency in the underlying model. Furthermore, this would not address potential issues with U-shaped decision boundaries.

I am not arguing here that this 1-dimensional mixture model is the optimal approach for QC using these two measures. Indeed, I believe that the idea of the authors to use dependence between these two quality measures to produce more intelligent quality threshold is an excellent one. Rather, I am trying to demonstrate that the information added by the mixture of linear models rather than a simpler 1-dimensional mixture model is not necessarily an improvement, and is not intuitive to me when I consider two correlated measures of cell quality.

In Figure 1 below, I have illustrated what I would desire when jointly modelling two (somewhat) correlated QC measures - using dependence between the measures to define a threshold that decreases along both axes

of the data, rather than creating a non-linear decision boundary that accepts some cells with "worse" values for both quality measures over those with "better" values. I think that some form of two-component mixture model is ideal for this problem. In very brief experimentation I found that a non-parametric mixture model (see (Chauveau and Hoang 2015)) seems to provide a relatively consistent measure of quality, creating a decision boundary that does not lead to the unusual behaviour of miQC in boundary regions. It is of course likely that this would also demonstrate undesirable behaviour under other circumstances.

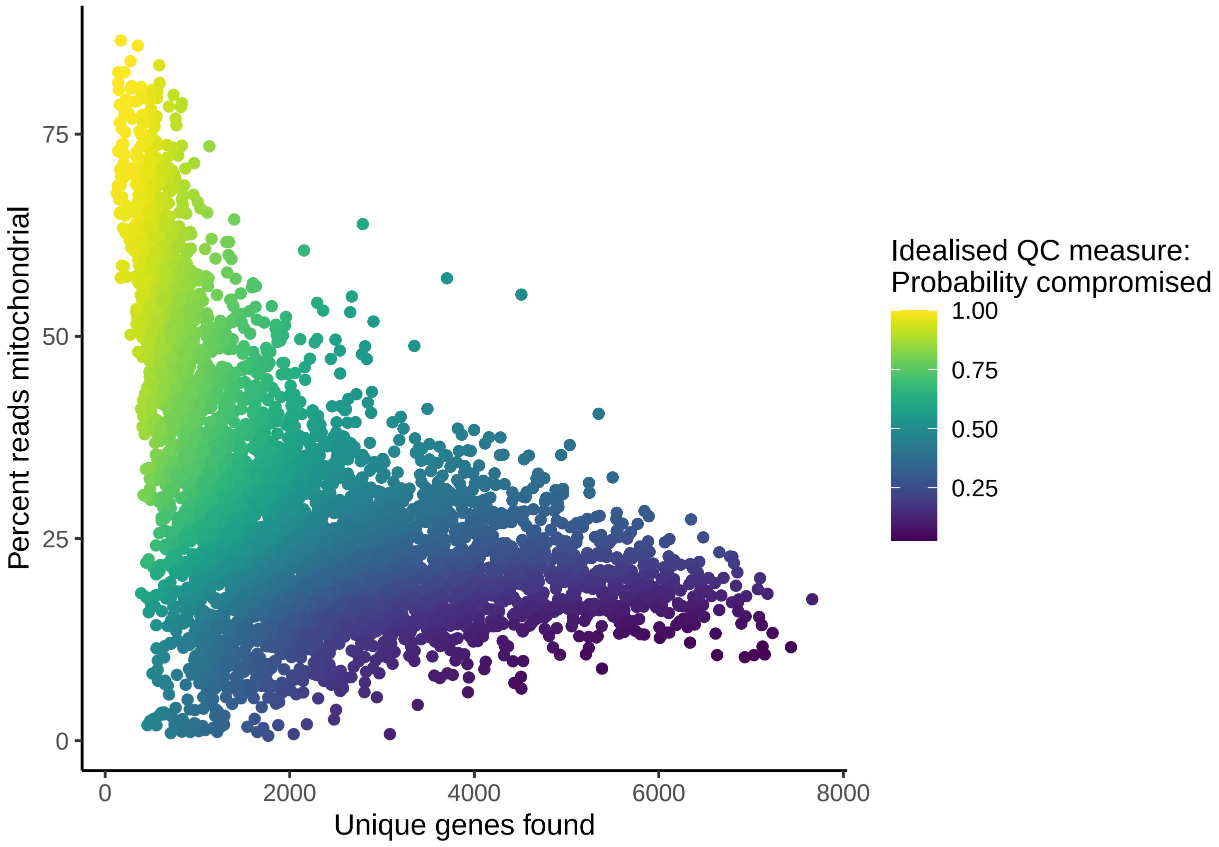

Figure 1: A QC score using both the number of genes detected and the mitochondrial proportion. This is generated using the median rank of each cell along both axes, and is presented simply for illustration purposes; it is not a viable QC score given that it does not provide any clear decision boundary.

Minor comments

- I would suggest the authors use a perceptually uniform colour scale, such as "viridis", in place of the gradient option currently used, when mapping continuous values to point colour.
- A general issue with the approach described is that it uses unconstrained linear models on constrained values - the predictor variable is constrained to $[0, \inf]$ and the response variable is constrained to $[0, 100]$. The model therefore allows impossible values (eg, negative mitochondrial proportion, negative number of unique genes). Could this be resolved by modelling these variables for example on a log-log scale, or by using a model that is constrained to the possible intervals of the data?

  This is a minor comment because I believe that a model that ignores this issue but empirically produces consistent and effective results would be preferable to a more principled approach that fails to do so.
- Author summary:

> “We developed the miQC package to jointly model both the proportion of reads mapping to mitochondrial (mtDNA) genes and the number of detected genes with mixture models”

This parses to me as “the number of detected genes with mixture models”. Perhaps this could be clarified with punctuation or by re-ordering the clauses in the sentence.

- Abstract:

  “Our software package is available in at” -> “Our software package is available at”
  Also suggest using Bioconductor link rather than github - authors have put the work in to having the package accepted and thus deserve the recognition and trust often afforded to Bioc packages.

- L14:

  > “One widely used QC metric to identify a compromised cell”

  Suggest rephrasing to “One QC metric widely used to identify a compromised cell is. . . ”. Similarly L31.

- L53:

  > For a given scRNA-seq sample, we model the cells using latent variable model with a true and unknown (or hidden) latent factor representing the two populations of cells.

  “using latent variable model” -> “using a latent variable model”

  Furthermore, is true necessary (or correct) here? It seems that this is a data-driven approach to making a qualitative judgement, with cells lying on a continuum of quality levels. To say that compromised/non-compromised is a binary latent variable is to imply that these are discrete states. However all of the contributing factors (rupture, stress, etc) are likely to lie on a spectrum.

  Perhaps this is a philosophical point, but to me it implies that there is a single “correct” answer to the problem of quality control, rather than a range of possible stringencies for different purposes.

- L70: As complexity has been explained before this point, you could make this easier to read by omitting the full explanation (eg, by simply saying “number of genes detected” as in abstract)

- L76:

  > “a wide range of the number of unique genes found”

  perhaps “a wide range of values for library complexity”?

- L80 (fig1 caption):

  > “the number of unique genes found”

  would prefer consistency between “found” and “detected”

- L188+ (fig 3 caption): “Color represent” -> “Color represents”

- L219+ (fig 4 caption): “A confusion matrix of what cells” -> “A confusion matrix of which cells are filtered by miQC using the different reference genomes, showing [. . .]” “larger number predicted intact cells” -> “larger number of [. . .]”

- L279:

  > “All datasets were processed using scater [23], as described in Section .”

  Reference to section is missing.

- L339:

  > “meaning the for the cells with a low library complexity”
  > “we labeled distribution with higher percent of cell counts mapping to mtDNA genes as the compromised cell distribution.”

  These seem like typos; it’s unclear to me what’s meant here.

- L404: “available as an user-friendly R package available” -> “available as a user-friendly R package available”

  suggest changing to “[…] package accessible at”

- L411: I believe rather than “Posterior likelihood” this should read “Posterior probability”

References

Chauveau, Didier, and Vy Thuy Lynh Hoang. 2015. “Nonparametric Mixture Models with Conditionally Independent Multivariate Component Densities.” https://hal.archives-ouvertes.fr/hal-01094837.
