# Supplementary material for: miQC: An adaptive probabilistic framework for quality control of single-cell RNA-sequencing data
Source: PLoS Comput Biol. 2021 Aug 24;17(8):e1009290. doi: 10.1371/journal.pcbi.1009290 (PMC8415599; doi:10.1371/journal.pcbi.1009290)
Supplement: Attachment — Submitted filename: Wang.pdf [file pcbi.1009290.s015.pdf]

Outline

This document contains a number of plots to demonstrate the properties of the miQC quality score when compared with a simpler approach. This was run in a directory that contains the `mito-filtering` repository.

1. Bivariate plot of quality measures with marginal density histograms.
2. Bivariate plot of quality measures, with colour representing miQC quality score.
3. Bivariate plot of quality measures, with colour representing quality score from a 1-dimensional mixture model.
4. Bivariate plot of quality measures, with colour representing which cells are rejected by miQC at the given probability threshold.
5. Bivariate plot of quality measures, with colour representing which cells are rejected by a 1-dimensional mixture model at the given probability threshold.
6. Bivariate plot of quality measures, with colour representing which cells are rejected (or not) by both of the methods (miQC and 1-dimensional mixture model).
7. Bivariate plot of proportion of mitochondrial reads against the total library size, with colour representing miQC quality score. This figure demonstrates that the behaviour of miQC is similar whether considering cellular complexity or library size as a secondary quality measure.
8. Bivariate plot of quality measures, with colour representing which cells are rejected by miQC at a more stringent probability threshold that may induce surprising behaviour.

Wang data

```
library("mixtools")
library("SingleCellExperiment")
library("scRNAseq")
library("scater")
library("flexmix")
library("splines")
library("BiocParallel")
library("biomaRt")
library("miQC")
library("viridis")
library("ggExtra")
theme_set(theme_classic())

set.seed(42)

## params references the params defined in the YAML frontmatter to the
## RMarkdown file.
data <- params$dataset

sce <- readRDS(paste0("mito-filtering/sce_objects/pre_filtering/", data, ".rds"))

if (is.null(sce$subsets_mito_percent)) {
    mt_genes <- grepl("^(mt|MT)-",  rownames(sce))
    stopifnot(any(mt_genes))
    feature_ctrls <- list(mito = rownames(sce)[mt_genes])

    sce <- addPerCellQC(sce, subsets = feature_ctrls)
}
```

```r
ggMarginal(plotMetrics(sce), type = "histogram")
```

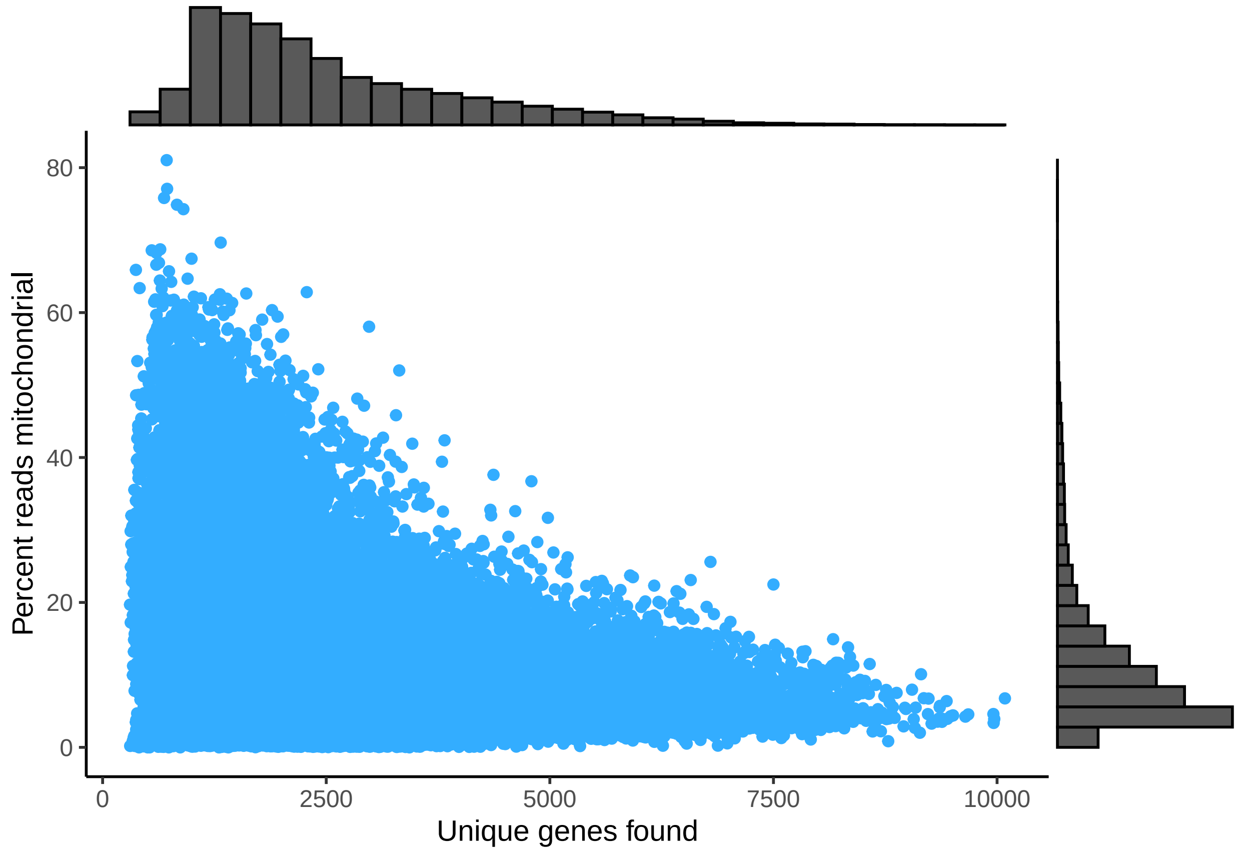

Figure 1: QC metrics with marginal distributions

```r
model <- mixtureModel(sce)
comp_component <- which.max(parameters(model)[1, ])
```

```r
means_mito <- quantile(sce$subsets_mito_percent, c(0.99, 0.1))
means_detected <- quantile(sce$detected, c(0.1, 0.9))

mm_mito <- normalmixEM(
    sce$subsets_mito_percent,
    lambda = 0.5,
    mu = means_mito
)
comp_component_mito <- which.max(mm_mito$mu)

lab <- labs(x = "Unique genes found", y = "Percent reads mitochondrial")
prob_threshold <- 0.75
prob_mito <- mm_mito$posterior[, comp_component_mito]
prob_miqc <- model@posterior$scaled[, comp_component]
```

```r
ggplot() +
    aes(
        sce$detected,
        sce$subsets_mito_percent,
```

```
        colour = prob_miqc
    ) +
    geom_point() +
    lab +
    scale_colour_viridis(name = "Probability\ncompromised")
```

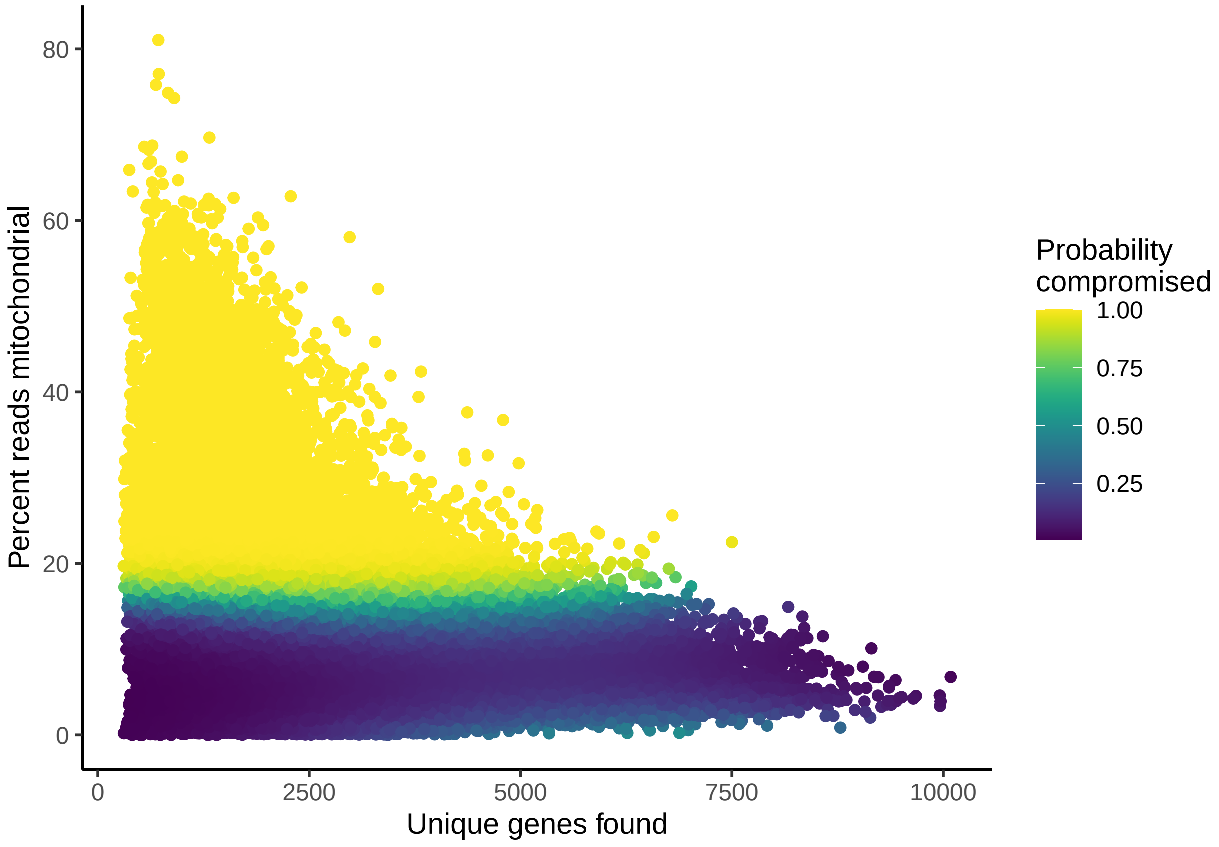

Figure 2: Probability compromised, miQC

```
ggplot() +
    aes(
        sce$detected,
        sce$subsets_mito_percent,
        colour = prob_mito
    ) +
    geom_point() +
    lab +
    scale_colour_viridis(name = "Probability\ncompromised")
```

```
ggplot() +
    aes(
        sce$detected,
        sce$subsets_mito_percent,
        colour = prob_miqc > prob_threshold
    ) +
    geom_point() +
    lab +
```

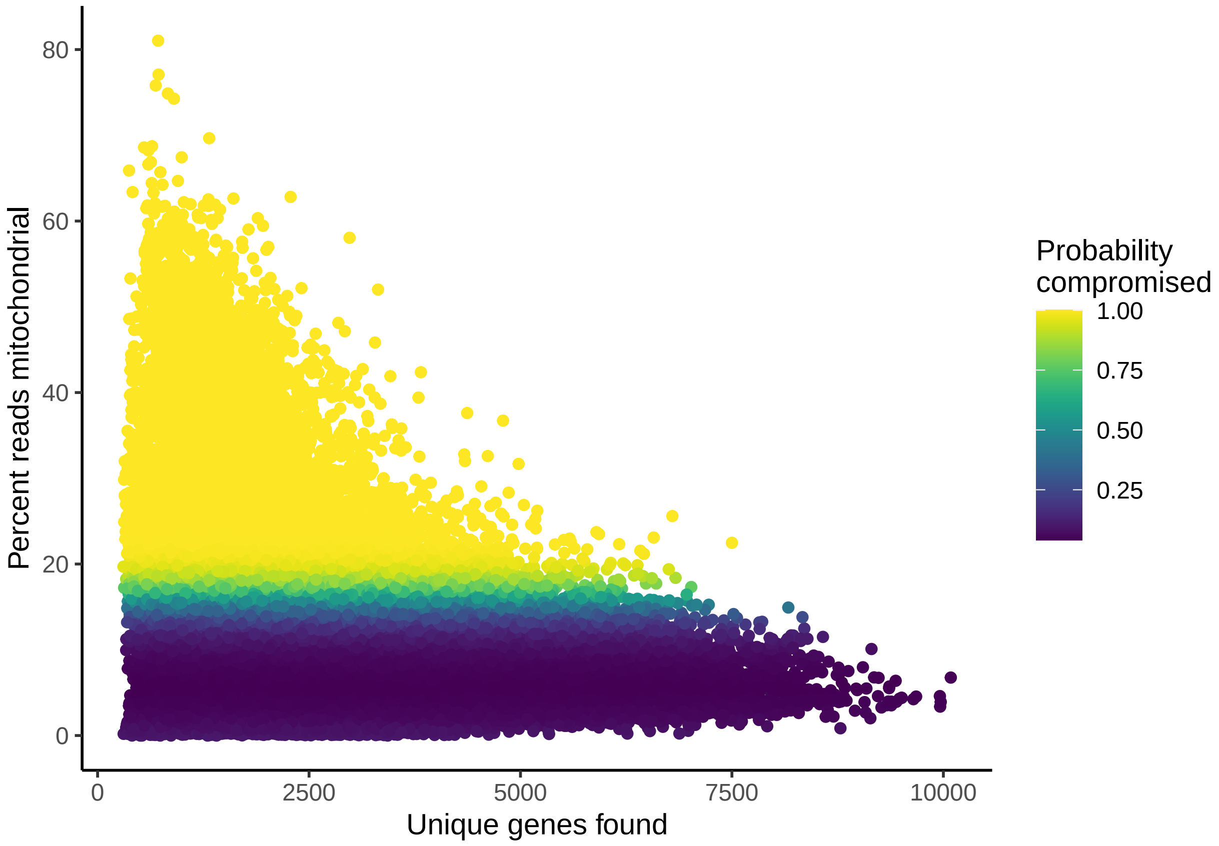

Figure 3: Prob compromised from normal mixture model using percent mito only

```
    scale_colour_manual(
        values = c("TRUE" = "firebrick", "FALSE" = "dodgerblue"),
        name = "QC fail, \nmiQC"
    )
```

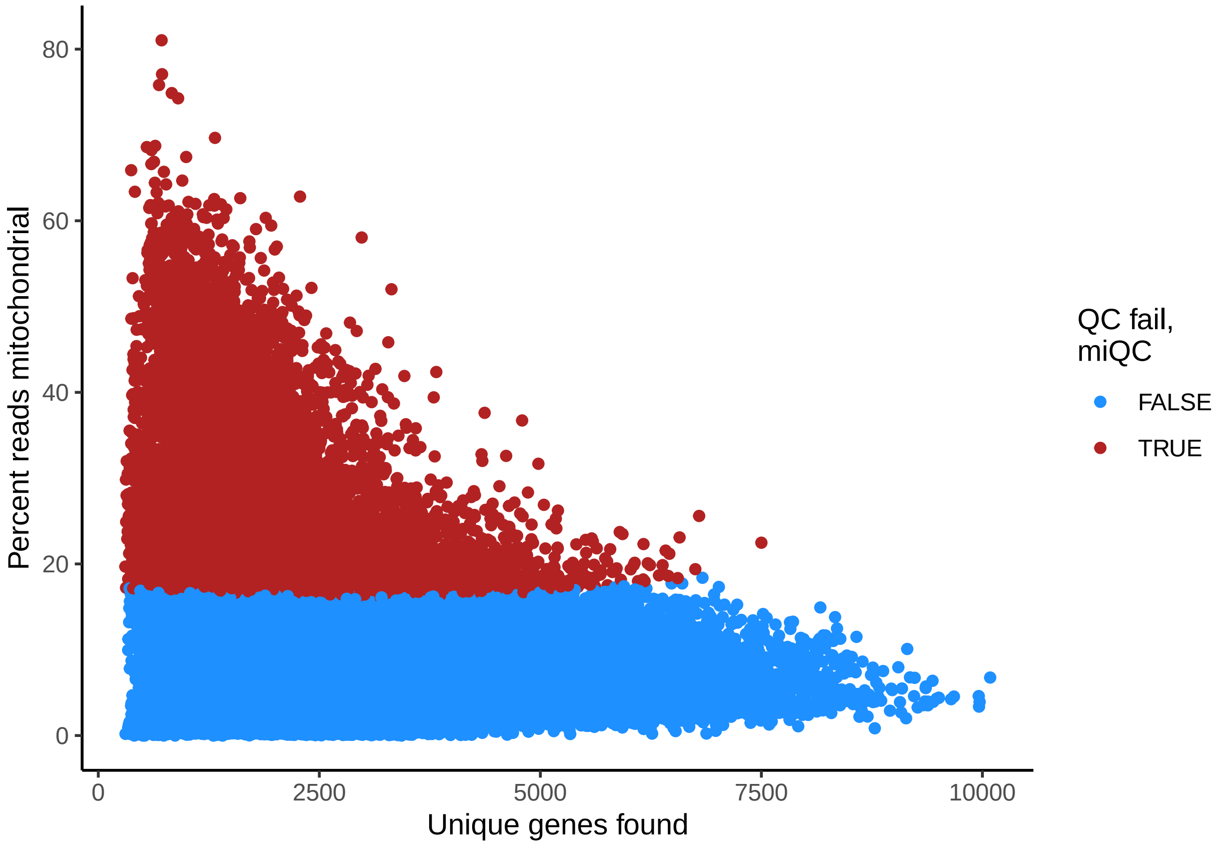

Figure 4: QC pass/fail with miQC

```
ggplot() +
    aes(
        sce$detected,
        sce$subsets_mito_percent,
        colour = prob_mito > prob_threshold
    ) +
    geom_point() +
    lab +
    scale_colour_manual(
        values = c("TRUE" = "firebrick", "FALSE" = "dodgerblue"),
        name = "QC fail, \n1D Gaussian\nmixture"
    )
```

```
ggplot() +
    aes(
        sce$detected,
        sce$subsets_mito_percent,
        colour = prob_mito - prob_miqc
    ) +
```

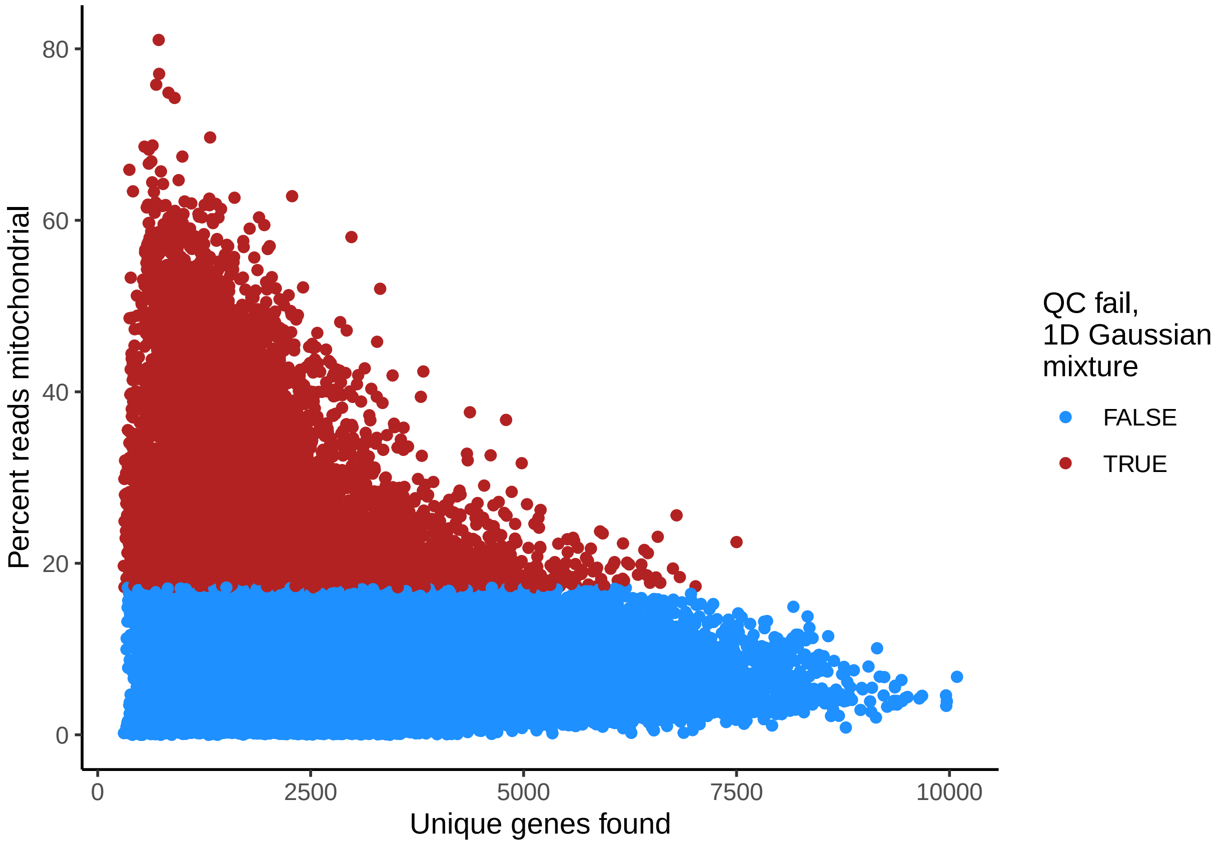

Figure 5: QC pass/fail with 1D mixture model

```
    geom_point() +
    lab +
    scale_colour_gradient2(
        low = scales::muted("blue"),
        high = scales::muted("red"),
        name = "Difference between \n1D normal mixture and \nmiQC"
    )
```

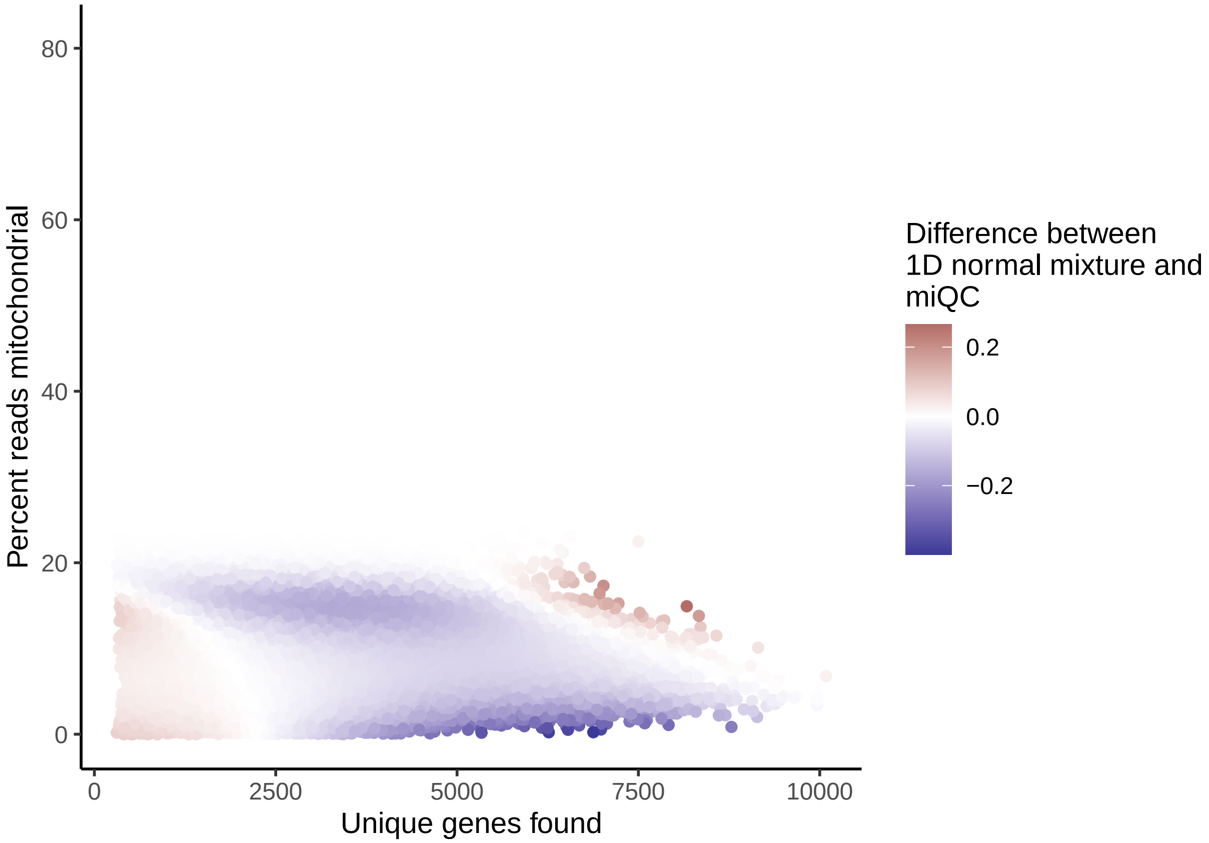

Figure 6: Comparison of posterior probs, 1D mixture and miQC

```
col <- paste(
    "miQC fail :", prob_miqc > prob_threshold, "\n",
    "1D normal mix fail :", prob_mito > prob_threshold, "\n"
)
col <- factor(col,
    levels = paste(
        "miQC fail :", c(TRUE, FALSE, FALSE, TRUE), "\n",
        "1D normal mix fail :", c(TRUE, FALSE, TRUE, FALSE), "\n"
    )
)
ggplot() +
    aes(
        sce$detected,
        sce$subsets_mito_percent,
        colour = col
    ) +
```

```r
    geom_point() +
    lab +
    scale_colour_brewer(name = NULL, drop = FALSE, palette = "Paired")
```

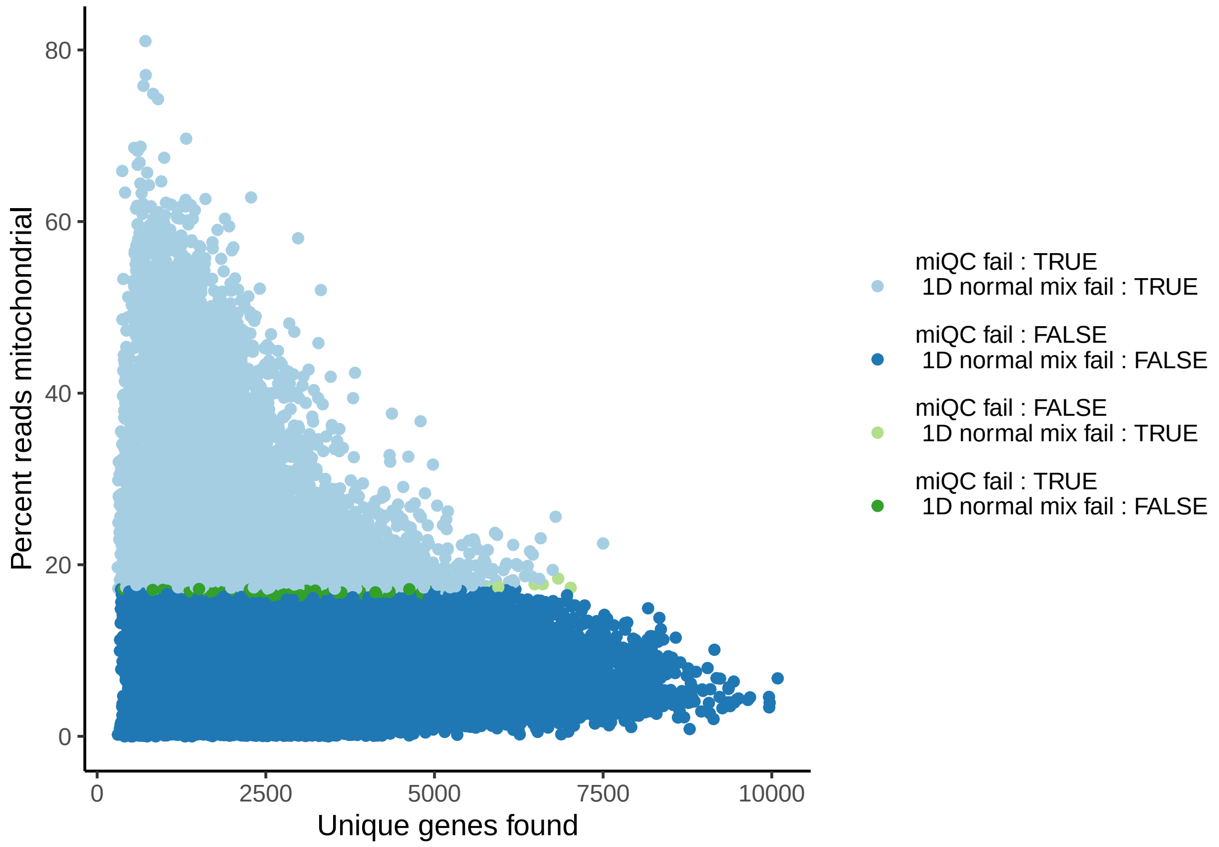

Figure 7: Comparison of pass/fail, 1D mixture and miQC

```r
ggplot() +
    aes(
        colSums(counts(sce)),
        sce$subsets_mito_percent,
        colour = prob_miqc
    ) +
    geom_point() +
    labs(x = "Total counts", y = "Proportion mito") +
    scale_colour_viridis(name = "Probability\ncompromised")
```

```r
ggplot() +
    aes(
        sce$detected,
        sce$subsets_mito_percent,
        colour = prob_miqc > (2/3)
    ) +
    geom_point() +
    lab +
    scale_colour_manual(
        values = c("TRUE" = "firebrick", "FALSE" = "dodgerblue"),
```

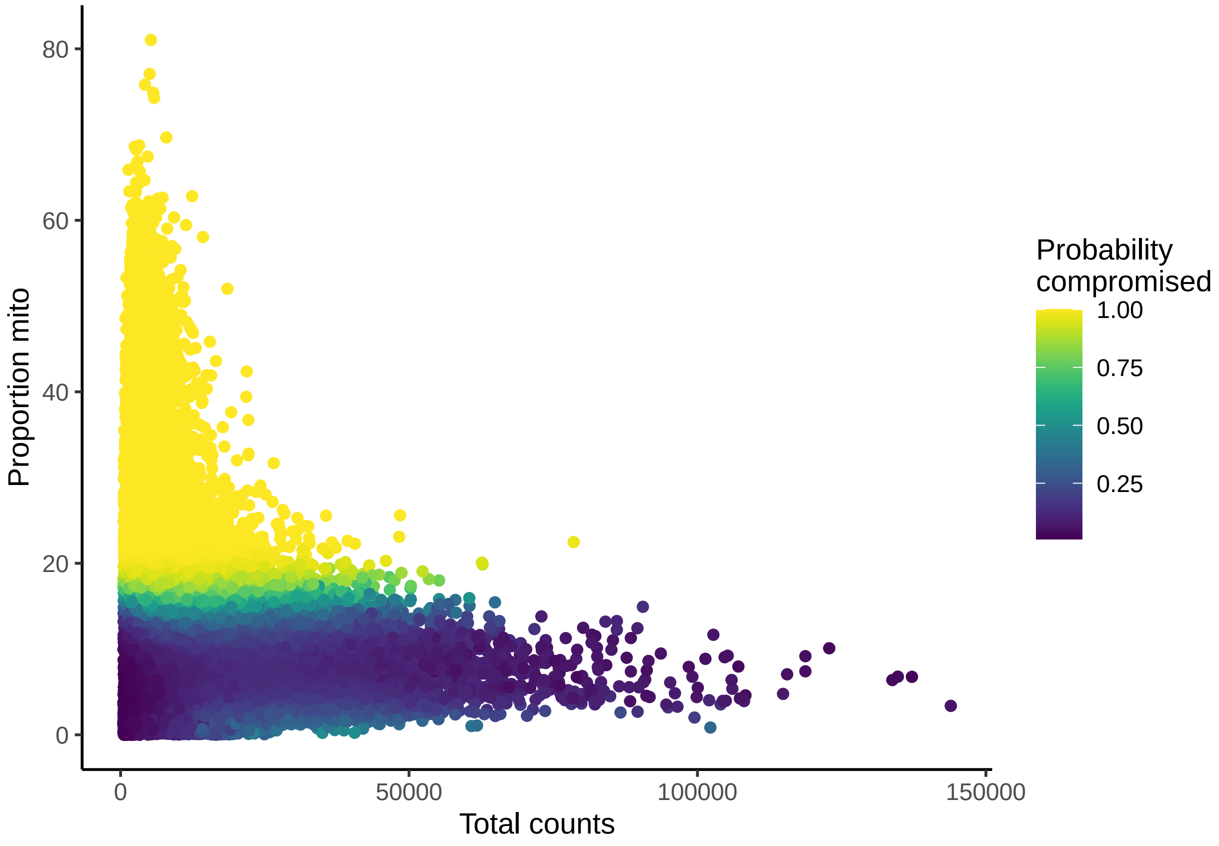

Figure 8: Prob compromised from miQC showing library size (total count).

```
        name = "QC fail, \nmiQC"
    )
```

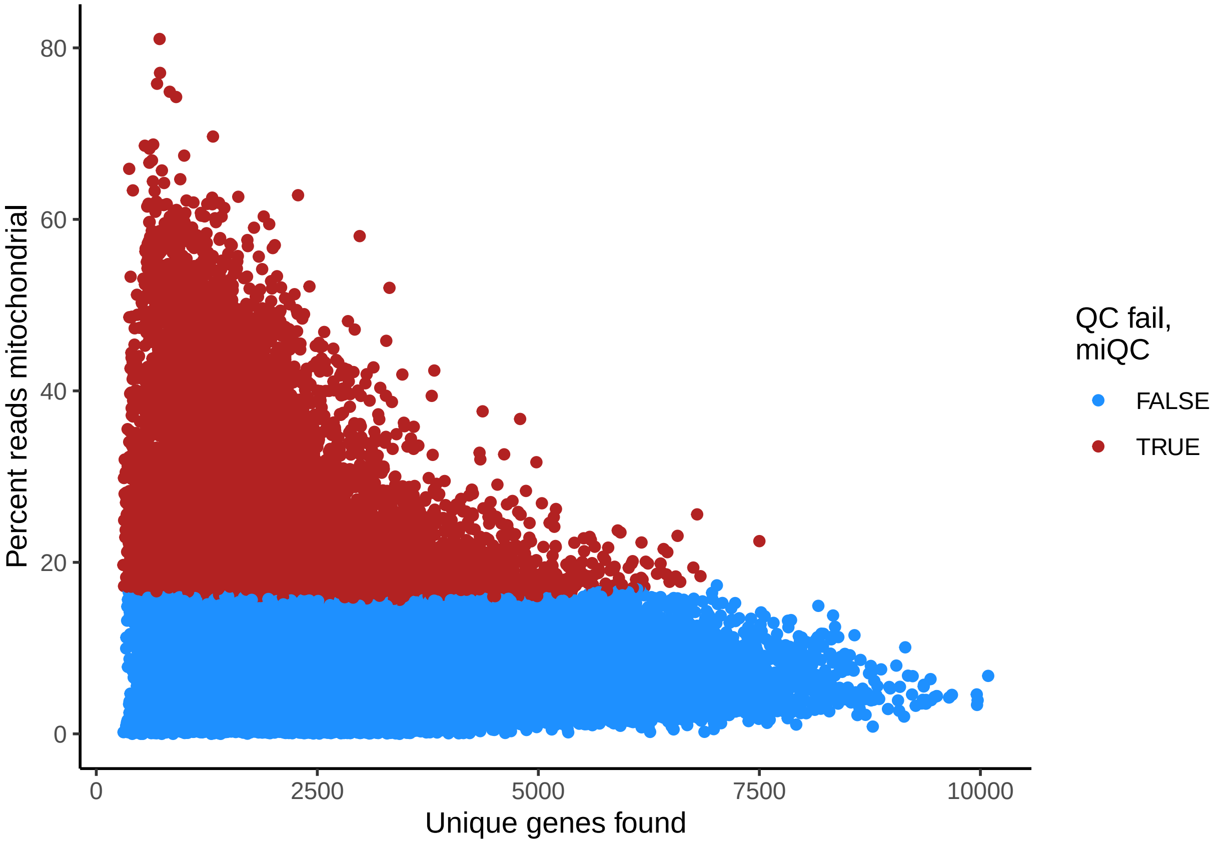

Figure 9: Cells rejected by miQC with a stringent posterior probability threshold of 2/3. This conceptually amounts to the evidence of cells being compromised as double the evidence of cells being non-compromised.
