# Supplementary material for: miQC: An adaptive probabilistic framework for quality control of single-cell RNA-sequencing data
Source: PLoS Comput Biol. 2021 Aug 24;17(8):e1009290. doi: 10.1371/journal.pcbi.1009290 (PMC8415599; doi:10.1371/journal.pcbi.1009290)
Supplement: Attachment — Submitted filename: Response to Reviewers.pdf [file pcbi.1009290.s017.pdf]

June 22, 2021

Dear Drs. Rattay and Ioshikhes,

Thank you for your consideration of our revised manuscript entitled 'miQC: An adaptive probabilistic framework for quality control of single-cell RNA-sequencing data' to *PLOS Computational Biology*. We appreciate the reviewer's constructive comments and in our enclosed revision we have addressed the comments raised from our original submission. We believe that the modifications have led to a stronger and more coherent manuscript.

Comments from all three reviewers (R1, R2, R3) were highly enthusiastic and supportive of the manuscript. Examples include: “an under-addressed component of the field of single cell computational biology” (R1), “commendable effort to devise an adaptive, data-driven quality measure for scRNAseq” (R1), “results are easily reproducible and their presentation of these materials is, in my view, exemplary” (R1), “miQC is a useful package to complement scater/scran” (R2), “method is simple enough to be transparent to the user (an advantage) and yet it does advance current (typical) practices in cell QC for scRNA-seq data” (R3), “authors convincingly demonstrate miQC's advantages over hard thresholding in several settings” (R3), “I found the software package easy to install and to use following the vignette provided with the package” (R3), “no concerns about it scaling reasonably to larger datasets” (R3), and “this method will be a useful and welcome addition to the Bioconductor ecosystem for scRNA-seq data analysis” (R3)

The majority of the critiques came from Reviewers #1 and #2, who asked for more motivation and explanation around our method compared to state-of-the-art. We have used these to improve both our manuscript and software and have updated our manuscript accordingly. All revised text is marked via latexdiff as recommended. Below you will find point-by-point responses.

Best wishes,

Stephanie Hicks

Stephanie Hicks
Department of Biostatistics
Johns Hopkins Bloomberg School of Public Health
615 N Wolfe St.
Baltimore, MD 21205

Reviewer reports:

Reviewer #1:

My main review comments are in the "miQC-review.pdf" file, generated from "review.Rmd". Other PDFs are supporting material to illustrate points made in that file. The Makefile defines rules to create each other PDF by rendering "comparison.Rmd" on different datasets. I have included the code used in case it helps, and also in case I have made some error(s) that affect the comments made in my review.

Summary

In this manuscript, the authors present an adaptive data-driven quality filtering threshold for scRNAseq data, based on two quality measures: the number of unique genes per cell, and the proportion of counts mapping to mitochondrial genes. The aim of this method is to identify "low-quality" cells that may bias the results of downstream analyses. This is a key component of every single-cell analysis, and is perhaps an under-addressed component of the field of single cell computational biology.

To this end, they use a finite mixture of regression models, where one component of the mixture represents "compromised" or low-quality cells, and the other component represents high-quality cells. The posterior probabilities from this mixture model are then used to classify cells as high-quality or low-quality. Using the dependence between the two quality measures, the authors aim to filter cells in a dataset-dependent way that can adapt easily to tissues containing varying numbers of high- and low-quality cells.

The authors make a commendable effort to devise an adaptive, data-driven quality measure for scRNAseq. Furthermore, I commend their presentation of their method as a reviewed Bioconductor software package, as well including the datasets and code used for analysis as a reproducible repository. Their results are easily reproducible and their presentation of these materials is, in my view, exemplary.

The authors place their method well in the context of the wider literature on the topic. Furthermore, their manuscript is well-organised and in general well-written and easily understood. However, at times there appear to exist discrepancies between the motivations they describe for a data-driven quality control measure and the behaviour of their approach on the data they present. This could be clarified by explaining the motivations behind their choice of model, by clarifying the reasoning why cells should be considered high- or low-quality, and/or by adapting their methodology to make it more consistent with their stated aims.

We thank the reviewer for their encouraging comments and helpful feedback. We have now carefully considered the suggestions and addressed them in the revised manuscript as we detail point-by-point below. Most importantly, we aimed to provide clarifications behind our choice of model, reasoning for why droplets might be considered as high or low quality, and a description of our now expanded methodology to make it consistent with our stated aims.

Major comments

While adaptive and data-driven approaches to quality control are both novel and desirable, it is not clear to me the motivation behind this specific approach. I am uncertain why one expects a different relationship between mitochondrial proportion and number of unique genes for "high-quality" and "low-quality" cells, as is implied by a mixture of linear models. It seems to me that the approach was chosen for simplicity based on the desire to identify cells in the top-left of the bivariate plots as low-quality and those in the remainder of the plot as high-quality. I think that this is fine conditional on the model producing results that are consistent with the view of these two measures as markers of cell quality.

We thank the reviewer for this comment. These two QC metrics (proportion of reads mapping to mtDNA genes and the library complexity) have been widely used <u>independently</u> to differentiate between the two types of cell libraries (compromised or "low-quality" and intact or "high-quality") during the quality control phase of the analysis; for example, in studies by Shekhar et al and Richard et al (references in paper) as well as the introductory vignette for seurat (https://satijalab.org/seurat/articles/pbmc3k_tutorial.html). Many previous studies have removed cells that have a high percent of reads mapping to mtDNA genes using either *ad hoc*, hard thresholds (e.g. 5%) or more data-driven thresholds (e.g. 3 or more median absolute deviations (MADs)). Note, if a user considered the library complexity on the *x*-axis and the mtDNA proportion on the *y*-axis, both the *ad hoc* threshold and MAD threshold result in a horizontal, linear decision boundary for classification. Alternatively, a data-driven, non-horizontal linear (or nonlinear) decision boundary can be used to be more inclusive to cell libraries with a higher library complexity (at a slight cost of higher proportion of reads mapping to mtDNA genes). To develop a non-horizontal decision boundary, we used this second piece of information, specifically the library complexity. This was our motivation behind this specific approach: namely to develop a non-horizontal, linear (or nonlinear) decision boundary to maximize and be inclusive of cell libraries that may have a slightly higher mitochondria percentage, but also have a higher library complexity, which is an established QC metric for "high-quality" cells. This is particularly important in the context of tissues, such as tumors, obtained during surgeries or other long-duration procedures where researchers want to ensure having sufficient statistical power to detect differences between cell types, which depends on having appropriate sample sizes of measured cells.

To summarize and incorporate this description into the manuscript, we have now expanded the text in the Introduction to include our motivation for the approach to jointly model these two QC metrics.

> ***"Current best practices filter on these QC metrics independently and often use uniform thresholds, sometimes species-dependent thresholds, which can lead to arbitrary linear classification boundaries (or cutoffs) that may not be appropriate for a given dataset. These cutoffs are often conservative, leaving only a small number of the remaining cells, which offers a non-representative sample of the tissue and constrains downstream analyses. Moreover, filtering on these metrics independently can fail to properly interpret legitimate biological reasons for a low score on a single QC metric, such as quiescent cell populations which will naturally have fewer***

unique genes expressed, or cells involved in respiratory processes which will naturally have higher mitochondrial expression [13].

Here, we propose an alternative approach to enable researchers to make data-driven decisions about which population a given cell comes from with respect to both mtDNA fraction and library complexity, which is adaptive across scRNA-seq datasets. We aim to create a data-driven linear (or nonlinear) decision boundary that can be more inclusive to cell libraries with high library complexity and higher proportion of reads mapping to mtDNA genes."

However, I think there are some inconsistencies between the stated motivation for an adaptive, data-driven method and how this method functions when applied. I think that these inconsistencies should be addressed before publication, and I hope they may lead the authors to consider adapting their method to address them. To illustrate some of these issues, I have included some PDF documents I generated using the authors' code and datasets. Each of these is was created by running an RMarkdown script using a selection of the datasets in the mito-filtering repository linked in the manuscript; primarily those examples used in the manuscript.

In these files, I have applied miQC, and a 2-component Gaussian mixture using only the proportion of mitochondrial counts. By comparing these approaches, I hope to illustrate some of the points I raise here. Figure numbering, captioning, etc is consistent throughout.

In the mixture model presented, the model component with the larger intercept is considered the component representing "compromised" cells, where the authors use the definition:

"a low-quality cell that is expected to have few unique genes represented and a high mitochondrial fraction".

This definition is consistent with the idea that the authors wish to select cells in the top-left region as "low-quality" and those outside as "high-quality". I will refer here for brevity to the "lower component" as the one representing high-quality cells and to the "upper component" as the one representing compromised cells.

Given the very small slope for the lower component in most examples presented, this lower mixture component is essentially a Gaussian distribution over values of mitochondrial proportion, with a fixed mean for all values of the proportion of genes detected. This seems sensible based on the marginal distribution of the data (Figure 1 in attached PDFs). The upper component, meanwhile, has a non-zero slope, representing a Gaussian with a mean that varies based on the number of unique genes detected. Therefore a natural question is: how does miQC compare to a 2-component Gaussian mixture on the proportion of mitochondrial reads, ignoring cellular complexity?

We thank the reviewer for addressing this. A Gaussian mixture model on fraction of mitochondrial reads is a simple data-driven model, and often gives meaningful results. We agree (as the reviewer mentions later) that integrating cell complexity information to the

model is valuable, which motivates the base settings of our model, but the one-dimensional model is a useful alternative. Therefore, we have now added it as an option in our miQC package, available through the `model_type="one_dimensional"` **parameter of the** `mixtureModel()` **function. We have added information on the one-dimension Gaussian mixture in the Results Section under the subtitle "Probabilistic classifications for scRNA-seq data quality using mixtures of linear models" and in the expanded Supplemental Figure 1.**

As seen in Figures 6 and 7 of the attached PDFs, these approaches are broadly similar, due in part to the similarity of the lower (non-compromised) mixture components of each model. Cells in the top-left region have almost identical posterior probability values and there are, in the main, few differences in the cells rejected and accepted by each approach. When differences arise, they do not appear favourable to miQC. miQC in some cases retains more cells with very low unique genes and relatively high MT proportion, while removing more cells with higher unique genes and lower MT proportion (see Figure 7 in 16030X4.pdf). Often, the decision boundary is slightly U-shaped (eg, Figure 4 in files 16030X2.pdf, 16030X4.pdf). This results in miQC giving a lower probability of being compromised to cells with "worse" scores on both of the quality measures being used.

This seems to me to be contrary to the stated motivations for this quality measure. Specifically, on line 84 the authors state:

> *"mitochondrial transcript content is expected to be relatively high also for intact ovarian cancer cells."*

Given that miQC removes cells with high MT proportion and high complexity, it appears that this would favour the removal of intact ovarian cancer cells with high mitochondrial proportion and high cellular complexity. The behaviour of miQC in these data therefore seems to conflict with the stated goal of retaining cells with higher MT proportion for cancer datasets.

We thank the reviewer for noting this concern. It is true that this method often accepts some cells that are farther left and higher up than other rejected cells, which is biologically counterintuitive. On the initial run of the manuscript, we concluded that the number of cells affected was usually small compared to the whole sample and therefore acceptable. However, in the latest version of the miQC software package (version 1.1.3, currently available through Bioconductor-devel or at https://github.com/greenelab/miQC) and in our updated manuscript, we now include an argument `enforce_left_cutoff` **(default is TRUE), which prevents this scenario from occurring. By identifying the excluded cell with the smallest percentage of mitochondrial reads and excluding anything with greater % mitochondrial reads and lower library complexity, no included cell can be worse in both QC metrics than an excluded cell. However, this parameter still allows for increased permissiveness for cells with relatively high mitochondrial content but high cellular complexity, like the intact ovarian cancer cells that the reviewer noted might have been excluded otherwise. The examples in the paper have been rerun with this new default parameter, and their corresponding figures and references in the text have been updated.**

The differences between the approaches are quite apparent when comparing the posterior probabilities between the two approaches (Figure 6 in attached PDFs). The right tail of the upper mixture component produces counterintuitive results. For example, in Figure 2 of 16030X2.pdf and 16030X4.pdf, miQC gives a higher probability of being compromised for with < 10% MT counts and c 4000 unique genes than cells with c. 25% MT counts and < 500 unique genes. Again, it seems counterintuitive that cells with excellent values for both quality measures should be considered probable low-quality cells, while cells with very poor values for both measures are not.

Outliers with very low proportion of mitochondrial reads would likely be considered outliers using this approach - this is visible in Figure 4 of Messmer.pdf. Furthermore, I would expect this inconsistency to be magnified if users supply more stringent posterior probability thresholds. Under this condition it could easily be the case that cells rejected by miQC would be located in all regions of the bivariate distribution of these two measures, rather than concentrated solely in the upper left region. For example, in Figure 9 in 16030X2.pdf and 16030X4.pdf, it's clear that a more stringent probability threshold of 23 causes cells with very low mitochondrial proportion and relatively high complexity to be rejected (c. 0% and c. 5000, respectively). The decision rule could be adapted for cases such as this by choosing to never reject cells with a proportion of mitochondrial reads that is lower than the intercept of the lower mixture component. However, it seems as though needing to adapt the decision rule after fitting the model points to some deficiency in the underlying model. Furthermore, this would not address potential issues with U-shaped decision boundaries.

We thank the reviewer for this excellent suggestion. The latest version of the miQC package now includes a parameter called `keep_all_below_boundary` (default is TRUE), which preserves the cells with a very low proportion of mitochondrial reads that the reviewer describes. We describe this conditional check in the Results Section under the subtitle "Probabilistic classifications for scRNA-seq..." of the paper.

I am not arguing here that this 1-dimensional mixture model is the optimal approach for QC using these two measures. Indeed, I believe that the idea of the authors to use dependence between these two quality measures to produce more intelligent quality threshold is an excellent one. Rather, I am trying to demonstrate that the information added by the mixture of linear models rather than a simpler 1-dimensional mixture model is not necessarily an improvement, and is not intuitive to me when I consider two correlated measures of cell quality.

In Figure 1 below, I have illustrated what I would desire when jointly modelling two (somewhat) correlated QC measures - using dependence between the measures to define a threshold that decreases along both axes of the data, rather than creating a non-linear decision boundary that accepts some cells with "worse" values for both quality measures over those with "better" values. I think that some form of two-component mixture model is ideal for this problem. In very brief experimentation I found that a non-parametric mixture model (see (Chauveau and Hoang 2015)) seems to provide a relatively consistent measure of quality, creating a decision boundary that does not lead to the unusual behaviour of miQC in boundary regions. It is of course likely that this would also demonstrate undesirable behaviour under other circumstances.

Figure 1: A QC score using both the number of genes detected and the mitochondrial proportion. This is generated using the median rank of each cell along both axes, and is presented simply for illustration purposes; it is not a viable QC score given that it does not provide any clear decision boundary.

We thank the reviewer for bringing our attention to this paper. A non-parametric model is a highly interesting idea. However, as the reviewer themselves mentioned, this metric would be difficult to implement or interpret in practice. A rank-based model, as the reviewer implemented in Figure 1 of their review, would require the user to decide what percentage of cells to discard, and it would be near-impossible to make consistent decisions across samples in an experiment, one thing that posterior probability over linear models allows for. In light of this, we have opted instead to stick to parametric models, specifically linear mixture models, with conditional checks to prevent undesired behavior. However, this could be a potential avenue for future work.

Minor comments
I would suggest the authors use a perceptually uniform colour scale, such as "viridis", in place of the gradient option currently used, when mapping continuous values to point colour.

Thank you for this suggestion. All figures using a gradient have been changed to "viridis".

A general issue with the approach described is that it uses unconstrained linear models on constrained values - the predictor variable is constrained to [0, inf] and the response variable is constrained to [0, 100]. The model therefore allows impossible values (eg, negative mitochondrial proportion, negative number of unique genes). Could this be resolved by modelling these variables for example on a log-log scale, or by using a model that is constrained to the possible intervals of the data?

This is a minor comment because I believe that a model that ignores this issue but empirically produces consistent and effective results would be preferable to a more principled approach that fails to do so.

While we agree with the reviewer that it is true that the unconstrained linear models can predict biologically impossible values, such as negative mitochondrial percentage, here we are using these models to calculate and filter on the posterior probabilities (not the direct predictor variable). This can be thought of quaintly as "which line is the cell closer to?" The biological possibility of the cell falling directly on one line or another is not relevant. Because the existing model produces empirically effective results, as the reviewer noted, and because transforming the QC metrics on a log-log scale would introduce other challenges, we have decided to keep the model as-is on this point. However, as future work one could explore a model that is appropriate for the range of the predictor variable [0, 100] to improve upon our proposed method in miQC.

Author summary: "We developed the miQC package to jointly model both the proportion of reads mapping to mitochondrial (mtDNA) genes and the number of detected genes with mixture models"

This parses to me as “the number of detected genes with mixture models”. Perhaps this could be clarified with punctuation or by re-ordering the clauses in the sentence.

This has been amended in the revised manuscript.

Abstract: “Our software package is available in at” -> “Our software package is available at”

This has been amended in the revised manuscript.

Also suggest using Bioconductor link rather than github - authors have put the work in to having the package accepted and thus deserve the recognition and trust often afforded to Bioc packages.

Thanks to the reviewer for mentioning this. At the point we submitted the original manuscript, the package was still under review at Bioconductor. Now that it has been accepted, we have changed to the Bioconductor URL in the revised manuscript.

L14: “One widely used QC metric to identify a compromised cell”

Suggest rephrasing to “One QC metric widely used to identify a compromised cell is. . . ”. Similarly L31.

The revised manuscript includes the suggested rephrasing.

L53: For a given scRNA-seq sample, we model the cells using latent variable model with a true and unknown (or hidden) latent factor representing the two populations of cells.

“using latent variable model” -> “using a latent variable model”

This has been amended in the revised manuscript.

Furthermore, is true necessary (or correct) here? It seems that this is a data-driven approach to making a qualitative judgement, with cells lying on a continuum of quality levels. To say that compromised/non-compromised is a binary latent variable is to imply that these are discrete states. However all of the contributing factors (rupture, stress, etc) are likely to lie on a spectrum.

Perhaps this is a philosophical point, but to me it implies that there is a single “correct” answer to the problem of quality control, rather than a range of possible stringencies for different purposes.

We thank the reviewer for raising this concern. We agree that the previous wording suggests discrete, binary categories which is misleading. In principle, the cell libraries are along a continuum, but for all practical purposes a data analyst needs to decide which cells to remove and which cells to keep for downstream analyses. This was our original intent for “two populations of cells”. However, we have now revised the sentence to “For a given

scRNA-seq sample, we model the cells using a latent variable model with a latent factor representing the degree of how compromised a given cell is.”

L70: As complexity has been explained before this point, you could make this easier to read by omitting the full explanation (eg, by simply saying “number of genes detected” as in abstract)

This has been amended in the revised manuscript.

L76: “a wide range of the number of unique genes found”

perhaps “a wide range of values for library complexity”?

This has been amended in the revised manuscript.

L80 (fig1 caption): “the number of unique genes found”

would prefer consistency between “found” and “detected”

This has been amended in the revised manuscript.

L188+ (fig 3 caption): “Color represent” -> “Color represents”

This has been amended in the revised manuscript.

L219+ (fig 4 caption): “A confusion matrix of what cells” -> “A confusion matrix of which cells are filtered by miQC using the different reference genomes, showing [. . .]” “larger number predicted intact cells” -> “larger number of [. . .]”

This has been amended in the revised manuscript.

L279: “All datasets were processed using scater [23], as described in Section .”

Reference to section is missing.

This has been amended in the revised manuscript.

L339: “meaning the for the cells with a low library complexity”

“we labeled distribution with higher percent of cell counts mapping to mtDNA genes as the compromised cell distribution.”

These seem like typos; it’s unclear to me what’s meant here.

This sentence has been revised in the manuscript and is hopefully more clear.

L404: "available as an user-friendly R package available" -> "available as a user-friendly R package available"

suggest changing to "[. . .] package accessible at"

This has been amended in the revised manuscript.

L411: I believe rather than "Posterior likelihood" this should read "Posterior probability"

This has been amended in the revised manuscript.

References

Chauveau, Didier, and Vy Thuy Lynh Hoang. 2015. "Nonparametric Mixture Models with Conditionally Independent Multivariate Component Densities." https://hal.archives-ouvertes.fr/hal-01094837.

<u>Reviewer #2</u>:

The authors propose a probabilistic mixture model called miQC that simultaneously models the proportion of reads mapping to mitochondrial genes (mtDNA) and the number of detected genes (nUMI). As the authors point out, the current "state of the art" is to set arbitrary thresholds on the distributon of mtDNA and nUMIs and there is a need for a multi-dimensional probabilistic model for QC of scRNA-seq data. The use of posterior probabilities to predict low-quality cells that miQC provides is handy, but the method is overall too simplistic and does not convincingly show improvement over current approaches. I think miQC is a useful package to complement scater/scran, but I do not think it delivers a sufficient advance to warrant publication as an article in PLOS Computational Biology. I am sorry that I cannot provide a more positive response and I hope my comments below are useful to the authors:

We thank the reviewer for their helpful feedback. We hope our modifications have addressed the reviewer's concerns in the revised manuscript. We provide a point-by-point response below.

Major comments:
- The authors state that "Current best practices use all these QC metrics independently and commonly use uniform thresholds". I do not think this is true. Just looking at the vignettes of (arguably) the two most commonly used packages for quality control of scRNA-seq (Seurat and scran/scater), both of them include scatterplots of mtDNA and nUMI to select low quality cells (https://bioconductor.org/packages/release/bioc/vignettes/scater/inst/doc/overview.html, https://satijalab.org/seurat/articles/pbmc3k_tutorial.html). As I mentioned above, a posterior probability is preferable over setting a threshold manually, but in practice, if using only two QC

dimensions these two approaches are almost equivalent (as shown in Fig1D, where one could simply set a threshold of ~30 mtDNA content).

We have clarified our language in the highlighted sentence, it now reads "Current best practices filter on these QC metrics independently and often use uniform thresholds." This better reflects the status quo as shown in tutorials the reviewer linked to. While the two metrics are often visualized together, the status quo is for users to employ thresholds that are applied independently, i.e. remove any cell with >5% mitochondrial reads or outside of 200-2500 unique features. The bivariate relationship between mitochondrial percentage and library complexity is not considered in making filtering decisions, for instance allowing for more mitochondrial percentage in the case of high library complexity as our model does with our new `enforce_left_cutoff` parameter (default is TRUE) implemented in miQC (version 1.1.3, currently available through Bioconductor-devel or at https://github.com/greenelab/miQC).

As to the reviewer's second point, that a two-dimensional model produces similar results, in our analyses we find that integrating the cell complexity information is valuable, particularly in order to preserve cells with high library complexity that may have high mitochondrial content as a reflection of their actual biology, not a technical artifact. We have, however, included an option in our miQC package to run a one-dimensional Gaussian mixture model on mitochondrial fraction to create a data-driven threshold. This option is noted in the Results Section under the subtitle "Probabilistic classifications for scRNA-seq data quality using mixtures of linear models" and visualized in the expanded Supplementary Figure 1.

- mtDNA content and nUMI are the two QC variables most commonly used in practice and the authors were right to choose them as input for their model. However, if one aims to use a latent variable model to define low quality cells I would include additional measurements in the model such as the fraction of features account for ~50% of the reads, the ribosomal fraction, etc. Most of these variables are highly correlated, but a latent variable model approach should be able to exploit such covariation patterns to provide better probabilistic decisions for low-quality cells.

We thank the reviewer for the suggestion to incorporate additional variables in the model, such as the ribosomal fraction. We explored this suggestion, but ran into several challenges, most importantly, the limitation of plotting in 3D space, which we use as a sanity check of the reasonableness of the distributions before filtering with the posterior probability. However, our goal for miQC is to provide the most options for an end user to implement a model that makes sense for their data and downstream experimental needs, while still making recommendations for what model will be simplest and most effective for most use cases. Therefore, we have included a paragraph in the Discussion explaining to a user how they can incorporate additional variables, such as ribosomal fraction, into their model using flexmix. However, for the purposes of miQC, we have decided to stick to the two most commonly used QC metrics, library complexity and mitochondrial fraction, for the base model.

- In Fig5 the authors compared miQC to the "the standard (?) approach of using a uniform QC threshold of 10% of cell counts mapping to mDNA genes". I do not think this is a fair comparison. The mtDNA QC threshold value is not universal and varies from experiment to experiment (even between samples from the same tissue).

The reviewer makes an excellent point about the non-uniformity of mitochondrial thresholds across tissues and experiments, and we agree the previous language was misleading. When we said "standard," we were referring to the recommendations of Osorio and Cai (DOI 10.1093/bioinformatics/btaa751), who indicated filtering on 10% mitochondrial fraction for human tissue samples should be the standard threshold. This has now been specified in the text describing Figure 5 and at other places in the paper that used the "standard approach" language. (It is also worth noting that the introductory tutorials for both seurat and scanpy, two widely used single-cell analysis suites, both recommend a 5% mitochondrial cutoff, creating another de facto standard QC threshold.)

- In practice, the QC thresholds are often adjusted after inspection of the latent manifolds (i.e. the UMAPs). Is there a way to incorporate the latent manifold as additional information for the probabilistic decision of low-quality cells? The package could automatically generate multiple UMAP plots under different QC thresholds and then try to come with an optimal threshold that maximises the number of intact cells but at the same time excludes clusters with compromised cells. If this approach is implemented efficiently, I think this could make a significant improvement in the current (tedious) QC pipelines

We thank the reviewer for this suggestion and we agree that the QC process is often iterative and can be tedious. However, our concern with what the reviewer is proposing is that the latent manifolds do not differentiate between technical and biological variation. If we were to implement what the reviewer is proposing (generate multiple UMAP plots under different QC thresholds), one underlying assumption would be that the proportion of reads mapping to mtDNA are technical (not biological), which is not always true. For example, in a recent bioRxiv preprint (https://doi.org/10.1101/2021.05.30.446342), researchers used scRNA-seq on adult human and mouse kidney cells, and they state how because "kidney has the second highest mitochondrial metabolic activity among organs in the human body (Mercer et al. 2011)", they used a permissive threshold of allowing all cells with less than 80% reads mapping to mtDNA to be used in downstream analyses. In this setting, the UMAP plots would all show clusters of cells with a large proportion of reads mapping to mtDNA and would be hence removed from downstream analyses. Our goal with miQC is to provide a data-driven threshold (before downstream analyses) that is flexible in whatever tissue or species is being investigated.

Reviewer #3:

This manuscript a new method and R software package to improve the quality control (specifically filtering out) of "compromised" (problematic or low quality) cells from scRNA-seq datasets prior to further downstream analysis. The idea itself is simple: use the two most important and most commonly used metrics for cell QC in scRNA-seq analyses (number of genes with non-zero

observed expression and percentage of expression from mitochondrial genes), but instead of applying hard thresholds as most people do, instead use a mixture model to identify populations of "compromised" and "good" cells, with a posterior probability of a cell being "compromised". The posterior probability can then be used as a threshold (the authors propose filtering out cells with a posterior probability of being compromised >75%) that combines the information about cell quality from the two underlying metrics. I view this paper as a relatively simple idea well executed. The method is simple enough to be transparent to the user (an advantage) and yet it does advance current (typical) practices in cell QC for scRNA-seq data. The authors convincingly demonstrate miQC's advantages over hard thresholding in several settings. They also offer a clear discussion of when miQC is not the best option, which is important information and appreciated.

As a user I found the software package easy to install and to use following the vignette provided with the package. The vignette is well written - clear and easy to follow. I was easily able to work through the code examples provided and it gave me the information I would need to use miQC in my own data analyses. The method is very fast on a dataset of 3005 cells, and I have no concerns about it scaling reasonably to larger datasets. Credit
to the authors for making their open-source code for the package and also code to reproduce the work presented in the manuscript easily available. The manuscript itself is clear, well-structured and well-written throughout. I have only a few minor comments and happily recommend publication of this work. I note that the authors plan to release the software package through Bioconductor, which I heartily support - this method will be a useful and welcome addition to the Bioconductor ecosystem for scRNA-seq data analysis.

We thank the reviewer for their encouraging comments and helpful feedback. We have now carefully considered the suggestions and addressed them in the revised manuscript as we detail point-by-point below.

Minor:
- Last sentence of abstract: "package is available in at https://..." -> delete "in"
- Suggest "extensive" on l7 is unnecessary
- l54: "using latent variable" -> "using a latent variable"
- l214: "This highlights..." - this what? Add a noun
- l223: "being being"
- l279: "Section ." - actual section label/number is missing in this sentence
- l336: "mixture two" -> "mixture of two"
- l340: "meaning the for the cells with a low library complexity" ?? clarify
- l340-41: "we labeled distribution" -> "we labeled the distribution"
- l342: "was" -> "were" (subject-verb agreement)

All of the above minor comments have been incorporated.

- Working through the vignette I did receive the following warning, which is not shown in the vignette:
> plotModel(sce, model) + viridis::scale_fill_viridis()
Warning message:

Removed 54 row(s) containing missing values (geom_path).
The authors will be able to determine if that's expected behaviour that is simply suppressed for clarity in the vignette or an issue that they wish to follow up on.

This is indeed expected behavior. The range of the plot is automatically scaled to (0, 100) but the compromised cell distribution line often descends below 0, causing ggplot2 to throw a warning. We have altered the function so that the warning should no longer be thrown. This should not affect the visualization or the actual analysis.
